# Hyperuricemia in Psoriatic Arthritis: Epidemiology, Pathophysiology, and Clinical Implications

**DOI:** 10.3389/fmed.2021.737573

**Published:** 2021-09-22

**Authors:** Cesare Tripolino, Jacopo Ciaffi, Piero Ruscitti, Roberto Giacomelli, Riccardo Meliconi, Francesco Ursini

**Affiliations:** ^1^Geriatric Medicine Unit, Department of Medical Functional Area, “San Giovanni di Dio” Hospital, Crotone, Italy; ^2^Medicine and Rheumatology Unit, Istituto di Ricovero e Cura a Carattere Scientifico (IRCCS) Istituto Ortopedico Rizzoli (IOR), Bologna, Italy; ^3^Rheumatology Unit, Department of Biotechnological and Applied Clinical Sciences, University of L'Aquila, L'Aquila, Italy; ^4^Unit of Allergology, Immunology, Rheumatology, Department of Medicine, Università Campus Bio-Medico Di Roma, Rome, Italy; ^5^Department of Biomedical and Neuromotor Sciences (DIBINEM), Alma Mater Studiorum University of Bologna, Bologna, Italy

**Keywords:** uric acid, gout, psoriasis, psoriatic arthritis, inflammation, cardiovascular

## Abstract

Psoriatic arthritis (PsA) represents the articular component of the systemic psoriatic disease and the extra-cutaneous disorder most frequently found in patients with psoriasis. Besides the articular involvement, PsA is associated with several metabolic abnormalities such as insulin resistance, hypertension, diabetes and hyperuricemia. Uric acid is the final product of purine metabolism and the etiological substrate of gout. Accumulating evidence highlights the emerging role of hyperuricemia as a major cardiovascular risk factor. Moreover, different studies evaluated the interplay between hyperuricemia and psoriatic disease, suggesting that individuals affected by psoriasis or PsA might present higher serum levels of uric acid and that hyperuricemia might affect severity of clinical manifestations and degree of inflammation in PsA patients. In this review, we focus on the bidirectional relationship between uric acid and PsA, analyzing how uric acid may be involved in the pathogenesis of psoriasis/PsA and how clinical manifestations of PsA and inflammatory mediators are affected by uric acid concentrations. Finally, the effects of anti-rheumatic drugs on uric acid levels and the potential benefit of urate-lowering therapies on psoriasis and PsA were summarized.

## Introduction

Psoriatic arthritis (PsA) represents the articular component of a complex clinical entity now recognized as “psoriatic disease.” Musculoskeletal involvement is reported in 20% of patients with psoriasis (Pso) (1) and encompasses a wide spectrum of manifestations including enthesitis, dactylitis, peripheral arthritis, and axial disease. Discrete clinical phenotypes (asymmetric oligoarthritis, symmetrical polyarthritis, distal interphalangeal joints arthritis, arthritis mutilans, spondylitis) have been historically identified although, in clinical practice, they frequently intersect each other (2). Beyond the joint involvement, PsA is burdened by a high prevalence of cardiometabolic comorbidities such as obesity, hyperlipidemia, diabetes, hypertension and hyperuricemia (3).

Uric acid (UA) is the end-product of purine metabolism. Recently, serum uric acid (SUA) gained popularity since an association with cardiovascular mortality and morbidity has been demonstrated (4). A number of studies investigated the interplay between hyperuricemia and Pso/PsA suggesting that subjects with these disorders also show higher levels of SUA or increased prevalence of hyperuricemia (5–21). The mechanisms behind these findings are not completely understood. Hyperuricemia was hypothesized to be a consequence of the accelerated cutaneous cells turnover observed in Pso or an epiphenomenon secondary to the metabolic disorders observed in Pso/PsA.

In the present review, we summarize the available literature regarding the association between Pso/PsA and hyperuricemia focusing on epidemiology, pathophysiology, and clinical implications.

Literature review was limited to published primary research, including basic science, cohort studies, intervention and observational trials, and review articles indexed in PubMed.

The following search terms were used: “psoriasis” OR “psoriatic arthritis” AND “urate” OR “uric acid” OR “monosodium urate” OR “gout” OR “urate-lowering agent.” The search was limited to articles written in English, with no date restriction. Title and abstract screening of all retrieved studies published up to 1st June 2021 was performed by two of the authors (CT and JC). Eligible articles proceeded to full-text assessment. As the intent of the review was narrative, inclusion was based on relevance, as deemed so by the authors, to one of the 5 subcategories of interest: (1) pathophysiology of uric acid in psoriasis and psoriatic arthritis, (2) uric acid levels in psoriasis and psoriatic arthritis, (3) uric acid levels and cardiovascular comorbidities in psoriatic arthritis, (4) effects of anti-rheumatic drugs on serum uric acid levels in psoriatic arthritis, and (5) effects of urate-lowering agents on serum inflammatory mediators.

## Uric Acid Metabolism and Biological Functions: A Brief Overview

UA is a heterocyclic compound of carbon, nitrogen, oxygen, and hydrogen derived from the exogenous and endogenous purine metabolism. Liver and gut, but also muscles, lungs, kidneys and the vascular endothelium represent the main sites of UA production. Normal SUA values range from 1.5 to 6.0 mg/dL in adult females and from 2.5 to 7.0 mg/dL in adult males (22, 23). Urate homeostasis is maintained by a finely regulated balance between production and excretion. Although kidneys are the main responsible for UA excretion (65–75% of UA elimination), also intestine plays a relevant role in UA metabolism (25–35% of UA elimination). Kidneys and intestine exert these activities through the presence of urate transporters on their surface. The primary urate transporters are urate transporter 1 (URAT1), located on the apical surface of proximal tubular cells, glucose transporter 9 (GLUT9), located on the basolateral membrane of the proximal tubule, and ATP-binding cassette subfamily G member 2 (ABCG2), located on both intestinal and renal cells surface (24). On the other hand, organic anion transporters (OAT1, OAT2, and OAT3) at the basolateral membrane, sodium-dependent phosphate cotransporters (NPT1 and NPT4) and multidrug resistance protein-4 (MRP4) at the apical membrane, mediate urate secretion. URAT 1, GLUT 9 and OAT4 are responsible for the tubular urate reabsorption (25).

Raised levels of serum UA derive from increased production, impaired elimination, or a combination of the two. With progress in understanding the pathogenesis, some authors proposed a new classification of hyperuricemia: renal overload type (including overproduction and reduced extra-renal excretion) and renal underexcretion type (26). Neoplastic diseases, Pso, obesity, alcohol consumption, and genetic disorders belong to the first group, whereas kidney disease, diuretics, and immunosuppressant agents are included in the second one (27).

UA formation involves a series of biochemical reactions that lead to the degradation of adenosine and guanosine. As first step, adenosine monophosphate is converted to inosine by a deaminase that removes an amino-group creating the inosine monophosphate. Afterward, it is transformed in inosine by a nucleotidase. Instead, guanine monophosphate is transformed in guanosine by the nucleotidase. Subsequently, an enzyme called purine nucleoside phosphorylase converts inosine and guanosine into hypoxanthine and guanine. The intervention of xanthine-oxidase will transform hypoxanthine in xanthine, whereas the guanine deaminase will convert guanine in xanthine. Finally, xanthine oxidase oxidizes xanthine, forming the uric acid molecule (23). Historically known as the causative agent of gout, uric acid has gained increased popularity in the last years for its double-faced nature as a risk and protective factor in various settings (28).

### Uric Acid as Protective Factor

SUA has a strong antioxidant effect and metal-chelating properties (22, 29), acting as a scavenger of plasma nitrogen radicals and reactive oxygen species, thus reducing the production of peroxynitrite (29, 30).

Furthermore, UA plays an important function as immune-stimulating agent especially in innate immune responses and type 2 immune responses (31, 32). Studies suggest that UA acts as a damage-associated molecular pattern after transformation in crystals of monosodium urate (33, 34). In this form it activates dendritic cells and enhances the innate immune system, behaving as an endogenous adjuvant (35, 36). Urate in its crystallin shape is phagocytized by monocytes or neutrophils causing the generation of pro-inflammatory cytokines such as IL-1. Furthermore, the internalization of UA crystals by leukocytes leads to production of free radicals, release of cathepsin B and activation of inflammasome (37, 38).

Interestingly, higher SUA levels have been hypothesized to play a protective role in the development of some neurological disorders such as dementia, multiple sclerosis, Alzheimer's disease and Parkinson's disease (39–42). In a recent meta-analysis involving 5,575 participants, Zhou et al. demonstrated a significant inverse association between SUA and Alzheimer's disease or Parkinson's disease (43). Moreover, the authors found a linear dose-response relationship between UA values and risk of dementia. In a retrospective analysis involving 1,166 subjects with ischemic stroke, higher UA levels proved a significant protective role in preventing negative neurological outcomes in male patients (44). The reasons behind these findings are not fully elucidated but it has been speculated that the antioxidant and metal-chelating properties of UA exert a neuro-protective effect on brain function and cognitive decline (45).

### Uric Acid as Risk Factor

Besides these beneficial effects, UA is also associated with several cardiovascular disorders (46). In the development of atherosclerotic lesions, UA up-regulates inflammatory signal pathways and promotes the pro-inflammatory response of M1 macrophages inhibiting the anti-inflammatory response of M2 (47). Moreover, hyperuricemia leads to development and progression of atherosclerosis through endothelial dysfunction. Intracellular UA reduces nitric oxide bioavailability impairing the activity of endothelial nitric oxide synthase and nitric oxide production (48). A role of UA has also been postulated in insulin resistance. Intracellular UA increases reactive oxygen species (ROS), which in turn cause β-cell apoptosis. Furthermore, due to the stimulation of inducible NO synthase (iNOS) gene expression, UA favors nitric oxide overproduction that leads to β-cell dysfunction and apoptosis and reduces glucose-stimulated insulin secretion (49).

Hyperuricemia is implicated in the development of hypertension and chronic kidney disease. UA activates the renin-angiotensin system and inhibits nitric oxide synthesis, promoting endothelial dysfunction, sodium reabsorption and proliferation of vascular smooth muscle cells. Moreover, uric acid triggers systemic inflammation and secretion of pro-inflammatory cytokines leading to increased extracellular fluid volume and vascular resistances, worsening systemic hypertension. Similarly, increased UA levels cause vascular and tubulointerstitial alterations that facilitate development and progression of chronic kidney disease (50).

However, a direct cause-effect relationship between hyperuricemia and chronic kidney disease or cardiovascular disease has not been definitely determined since hyperuricemia is often associated with other cardiovascular risk factors such as obesity, metabolic syndrome and hypertension, which might contribute to cardiovascular morbidity. In 2017, Li et al. (46) conducted a review of systematic reviews, meta-analyses and Mendelian randomization studies, analyzing the correlation between UA and 136 unique health outcomes. The authors concluded that a definitive association exists only between UA and nephrolithiasis, whereas the link with conditions such as heart failure, hypertension, impaired fasting glucose or diabetes, chronic kidney disease and coronary heart disease was deemed highly suggestive.

## Pathophysiology of Uric Acid In Psoriasis and Psoriatic Arthritis

**Figure 1:**
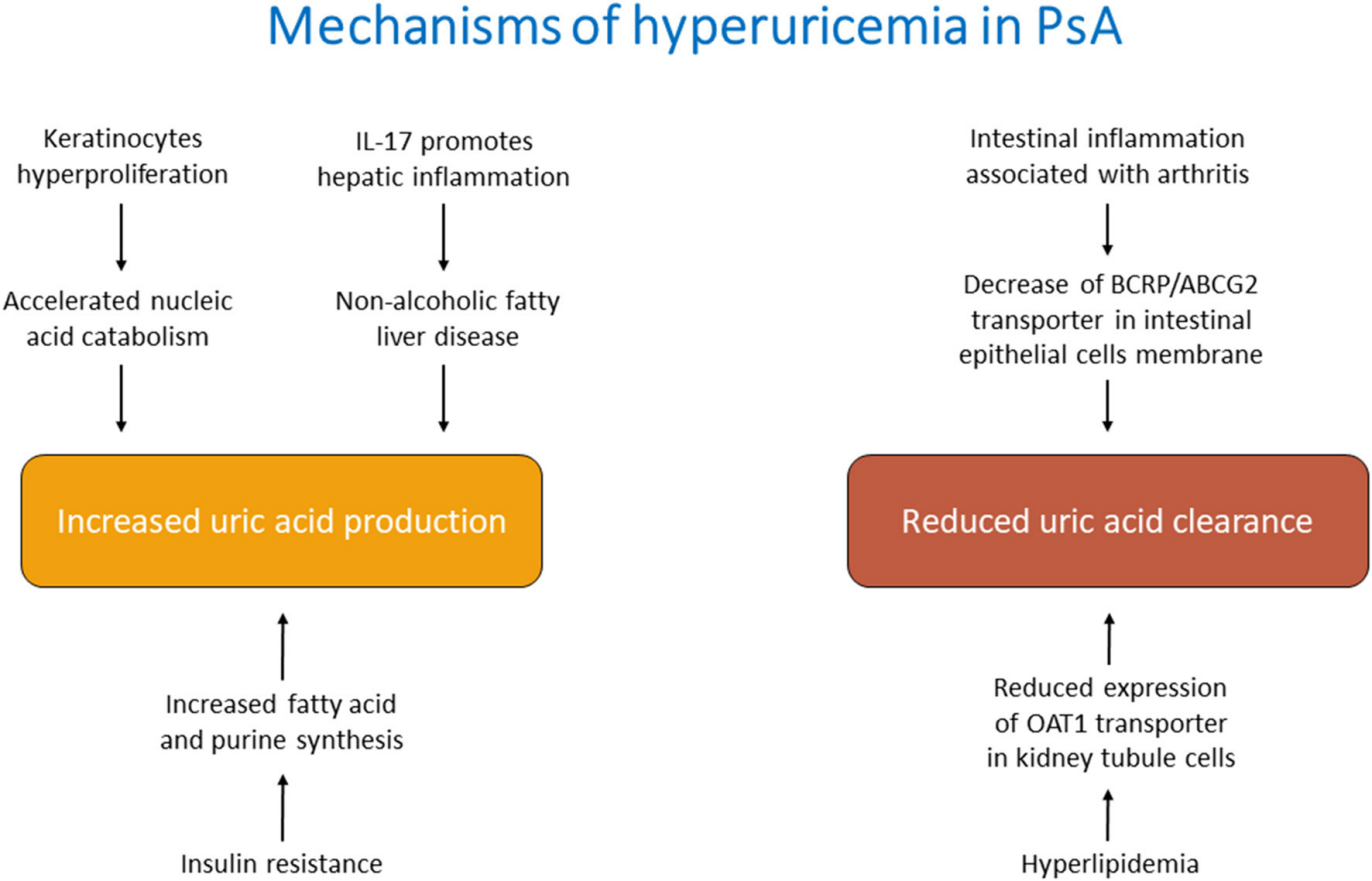
Main mechanisms of hyperuricemia in psoriatic arthritis.

The mechanisms behind hyperuricemia in Pso/PsA are still not fully elucidated and in part can only be hypothesized. High levels of SUA could be the consequence of an increased production, a reduced excretion or a combination of these factors (Figure 1).

It is plausible that, in the course of PsA, all these mechanisms contribute, favoring the development of hyperuricemia. It is also possible to distinguish *direct causes* of increased uric acid, related to the pathogenetic features of PsA, and *indirect causes*, linked to comorbidities or pharmacological treatment.

One of the recognized mechanisms leading to hyperuricemia in PsA is the increased cellular turnover characterizing this disorder (51). Indeed, the hyperproliferation of keratinocytes leads to accelerated nucleic acid catabolism and enhanced UA synthesis. However, hyperuricemia might also derive from an increased UA production in the liver. The overwhelming cytokines production in PsA, in particular IL-17, can affect the liver, leading to hepatic complications such as non-alcoholic fatty liver disease (52). Previous investigations found lower hepatic ATP production in hepatic steatosis (53, 54) that in turn causes increased production of UA from the hepatocytes (55).

Additional conceivable mechanisms might be related to the reduced renal and extra-renal clearance. Although the kidney represents the main actor of uric acid excretion, also other organs such as the intestine contribute to its balance. BCRP/ABCG2 is located at the apical membrane of small intestinal epithelial cells and it is involved in the UA secretion from blood into the intestinal lumen (56). A proportion of PsA patients might present evidence of clinical or microscopic inflammatory bowel disease, which might reduce the levels of these transporters and consequently impair UA homeostasis (57–59).

Hyperuricemia may be the consequence of the metabolic comorbidities associated with PsA: obesity, hypertension, insulin resistance or diabetes (60). In obese individuals, hyperuricemia is the result of both impaired excretion and overproduction of UA (61). In hyperlipidemia, the major mechanism causing hyperuricemia is the altered lipid metabolism. Indeed, the increased production of triglycerides lessens the expression of OAT1 in the kidney, leading to a reduced excretion of UA (25).

Again, the mechanism of overproduction of UA during insulin resistance states lies in the increased fatty acid synthesis in the liver that causes *de novo* purine synthesis and accelerated UA production (61–63). Furthermore, insulin stimulates the renal UA transporters leading to hyperuricemia (64).

**Figure 2:**
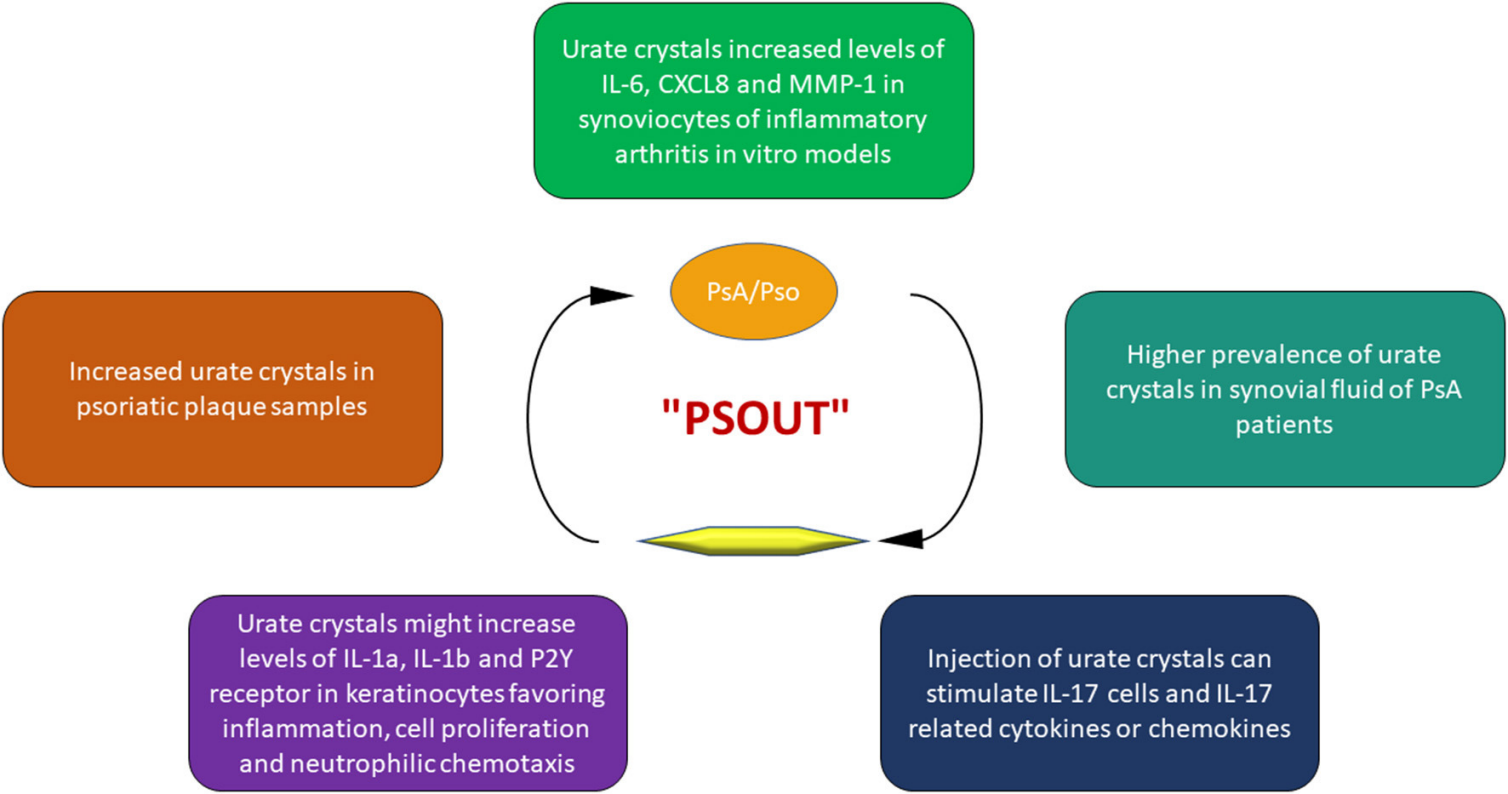
Key points of the interplay between uric acid and psoriatic disease.

Beyond the mechanisms underlying hyperuricemia, an intricate interplay between Pso, PsA and UA has been recognized (Figure 2), to the point that some authors proposed the term “psout” (65).

Indeed, it seems that UA could initiate and progress the alterations of PsA. The first observation about this issue dates back to 1981 when Goldman (19) found a high prevalence of urate crystals in samples of Pso plaques.

In a retrospective study, Oliverio et al. (66) analyzed the synovial fluid of patients with various articular disorder such as rheumatoid arthritis, osteoarthritis, gout, and PsA. The results of this investigation demonstrated a higher prevalence of urate crystals in PsA patients. Furthermore, the injection of urate crystals *in vivo* leads to the production of Th17 cells and Th17-related inflammatory cyto-chemokines, such as IL-17, one of the main mediators in the pathogenesis of PsA (67).

Urate crystals are able to stimulate human keratinocytes to produce several inflammatory cytokines and chemokines, in particular IL-1α and IL-1β, involved not only in gouty flares but also in the pathogenesis of PsA (68). Moreover, UA increases the expression of purinergic receptors P2Y on keratinocytes, stimulating cell proliferation (69), and promoting the production of IL-8/CXCL8, promoting neutrophil chemotaxis (70, 71). Finally, *in vitro* studies on synoviocytes from healthy and rheumatoid arthritis subjects, demonstrated that monosodium urate crystals were able to increase the production of inflammatory mediators such as IL-6, CXCL8 and matrix metalloproteinase-1 (72, 73).

Taken together, these evidences suggest that hyperuricemia doesn't represent a simple epiphenomenon in the course of PsA, but may play an important role in its development and progression.

## Uric Acid Levels in Psoriasis and Psoriatic Arthritis

A summary of the included studies is reported in Table 1. The association between Pso and hyperuricemia was first described in a pioneering article by Hermann in 1930. Among 140 Pso patients, 31% had raised SUA levels (5). These data were further confirmed in early subsequent research, showing a prevalence of hyperuricemia of 30–50% in Pso (6, 7), while more recent estimates reported a figure of 15% (8). Other studies investigated mean levels of SUA rather than prevalence of hyperuricemia, confirming that Pso patients present significantly higher SUA concentrations than non-Pso controls (9, 10, 12, 15).

**Table 1:** Characteristics of studies included in the evaluation of the association between serum uric acid levels and psoriasis or psoriatic arthritis.

**Author**	**Study design**	**Sample description**	**Outcome of interest**	**Main findings**
Baumann and Jillson (5)	Cross-sectional	140 patients with Pso or PsA	Prevalence of hyperuricemia	Hyperuricemia was present in 44 (31.4%) Pso patients, most often in those with PsA.
Eisen and Seegmiller (6)	Cross-sectional	38 Pso patients (18 males, 20 females)	Prevalence of hyperuricemia	Hyperuricemia was present in 19 (50%) Pso patients.
Steinberg et al. (7)	Cross-sectional	167 Pso patients (98 males, 69 females)	Prevalence of hyperuricemia	Hyperuricemia was present in 47 (48%) of male patients and in 19 (27%) of female patients.
Kamiya et al. (8)	Retrospective cohort	15,287 Pso patients (9,989 males, 5,298 females)	Prevalence of hyperuricemia	Hyperuricemia was present in 15.1% of patients (19.1% of males and in 6.3% of females).
Scott and Stodell (74)	Prospective cohort	41 Pso patients, 41 contact dermatitis, 41 healthy	Difference in SUA levels	No significant difference between the three groups nor associations between SUA and Pso extent.
Alpsoy et al. (9)	Prospective cohort	60 Pso patients and 50 healthy controls	Difference in SUA levels	Mean SUA concentration was significantly higher in Pso than in controls.
Isha et al. (10)	Prospective cohort	25 Pso patients, 25 healthy controls, 25 patients with skin disorders other than PSo	Difference in SUA levels	Mean SUA concentration was significantly higher in Pso than in the other two groups.
Kwon et al. (11)	Retrospective cross-sectional	198 Pso patients	Difference in SUA levels between Pso patients and general population. Assess correlation between Pso characteristics and SUA levels.	No difference in SUA levels between Pso patients and general population. Significant correlation between SUA levels and PASI (*p* < 0.05). Significantly higher PASI in hyperuricemic group than in normouricemic group. PASI was an independent risk factors for hyperuricaemia (*OR* =1.10; *p* =0.03).
Gisondi et al. (12)	Prospective cohort	119 Pso patients and 119 healthy controls	Prevalence of hyperuricemia and difference in SUA levels.	Higher SUA levels in Pso patients independently of gender). Asymptomatic hyperuricemia was found in 19% of Pso patients compared with 7% of controls (*p* < 0.001). Pso patients with PASI score ≥10 had higher SUA levels than patients PASI < 10. Pso was the strongest predictor of hyperuricemia (*OR* = 3.20; *p* < 0.01) independently of other variables.
Gisondi (75)	Prospective cohort	338 Pso patients	Assessment of characterics of Pso patients.	Hyperuricemia was present in 20%. SUA levels were higher in obese patients than in non-obese. SUA levels were not significantly correlated with PASI.
Ataseven et al. (76)	Prospective cohort	56 Pso patients and 33 healthy controls	Prevalence of hyperuricemia and assessment of characteristics of Pso patients.	No difference in SUA levels between groups. In Pso patients PASI showed a significant positive correlation with SUA (*r* = 0.27; *p* = 0.046).
Li et al. (13)	Meta-analysis	Total of 1,644 Pso patients and 27,393 controls	Association berween Pso and SUA levels.	SUA levels significantly higher in Pso patients than in controls. In the studies considering presence of hyperuricemia as a dichotomous variable, significantly higher prevalence in Pso patients than in controls (pooled RR = 2.18; 95% CI 1.29–3.68; *p* = 0.004).
Lai and Yew (14)	Population-based cross-sectional study	297 patients with Pso and 1,493 patients with hyperuricemia in a cohort of 11,282	Risk of hyperuricemia in Pso patients compared with controls.	Patients with Pso were at an increased risk of having hyperuricaemia (OR = 1.37; 95% CI 1.01–1.86; *P* = 0.04). The association was not significant after adjusting for confounders in multivariate regression analysis. No association between Pso severity and risk of hyperuricemia.
Solak et al. (15)	Case-control study	199 Pso patients and 54 healthy controls.	To compare SUA levels between Pso patients and general population.	SUA levels higher in patients with Pso compared to healthy controls. SUA levels did not correlate with the cutaneous extent of Pso.
Dehlin et al. (77)	*Post-hoc* analysis of randomized control trials	1,042 patients with Pso and 204 with PsA.	To determine the impact of Pso activity on SUA levels.	The degree of skin involvement showed a statistically significant, although modest, association with SUA levels (R^2^ = 0.014; p < 0.0001) at baseline. After 12 weeks of treatment, improvement in PASI score resulted in a decrease of SUA levels (R^2^ =0.014; p < 0.0001 univariately).
Kuo et al. (78)	Case-control study	39,111 patients with incident gout and 39,111 controls	To determine the burden of comorbidities in patients with gout at diagnosis and the risk of developing new comorbidities post diagnosis.	Patients with gout had increased hazard ratios for Pso (HR =1.53, 95% CI 1.37–1.74; *p* < 0.05). Pso, in turn, increased the risk of having incident gout (OR = 1.32).
Lambert and Wright (79)	Cross-sectional observational	115 patients with PsA (52 men, 63 women)	To determine the prevalence of hyperuricemia in PsA.	Hyperuricemia was present in 7 (13.5%) men and 3 (5%) women. After comparing PsA patients with and without hyperuricemia, no differences in terms of disease activity or Pso extent were found.
Bruce et al. (80)	Prospective cohort	265 PsA patients	To determine the prevalence of hyperuricemia in PsA and to determine the influence of skin involvement on SUA levels.	The authors found an incidence of hyperuricemia close to 21%, while incidence of gout was 0.8%. No association between PASI score and SUA.
Merola et al. (81)	Prospective population-based study	27,751 men and 71,059 women	To determine the risk of gout in Pso.	In the subgroup analysis of participants with self-reported Pso, the multivariate-adjusted HR for gout were 1.79 (95% CI 1.30–2.47) in men and 1.63 (95% CI 1.17–2.27) in women. The multivariate HR were higher among men (HR = 2.72, 95% CI: 1.75, 4.25) than in women (HR = 1.40, 95% CI: 0.90, 2.19) with confirmed Pso. Patients with Pso and concomitant PsA had a high risk of incident gout (HR = 4.95, 95% CI 2.72–9.01).
Lai et al. (82)	Cross-sectional observational study	160 PsA patients	Hyperuricemia in PsA patients	Hyperuricemia was present in 31% of patients. In simple correlation analysis, hyperuricemia was associated with PASI score (*p* = 0.05) and Body Surface Area (*p* = 0.04).
AlJohani et al. (83)	Prospective cohort study	318 hyperuricemic PsA patients and 318 normouricemic PsA patients	Investigate the prevalence and characteristics of psoriatic patients with hyperuricemia and to determine the adverse effect of hyperuricemia on outcomes	Huperuricemic patients had longer disease duration of PsA (*p* < 0.001) and Pso (*p* < 0.001) and higher PASI scores (*p* = 0.006). Multivariate analysis showed an association between persistent hyperuricemia and disease duration of PsA (OR 1.073, 95% CI 1.028–1.113).
Tsuruta et al. (84)	Retrospective cohort study	55 subjects with PsA and 276 with Pso	To determine factors associated with development of PsA in subjects with Pso	Hyperuricemia was significantly more prevalent in patients with Pso and PsA than in the group with only Pso (22 vs. 9%; *p* = 0.01). In multiple logistic regression, hyperuricemia was a strong predictor of PsA development (OR = 4.18; *p* < 0.01).
Hu et al. (20)	Population-based case-control study	114,623 patients with gout compared with 114,623 subjects without gout	Investigate association between Pso, PsA, and gout	Higher prevalence of Pso (1.6 vs. 1.1%; *p* < 0.0001) and PsA (0.3 vs. 0.1%; *p* < 0.0001) in patients with gout than in controls. In multiple logistic regression, gout was significantly associated with Pso (OR 1.30, 95% CI 1.20–1.42; *p* < 0.001) and PsA (OR 2.50, 95% CI 1.95–3.22; *p* < 0.001).
Kaine et al. (21)	Retrospective observational	14,898 PsA patients and 35,037 matched controls	To investigate frequency and incidence of comorbidities in adult patients with newly diagnosed PsA	PsA patients showed a higher risk for gout (incidence rate = 1.28 vs. 0.66; HR = 2.03; 95% CI 1.75–2.36) compared with the control group.

*PASI, psoriasis area severity index; PsA, psoriatic arthritis; Pso, psoriasis; SUA, serum uric acid*.

In a meta-analysis, Li et al. (13) identified a remarkable difference in SUA levels between subjects with Pso and controls (mean difference 0.89 mg/dl, 95% CI 0.05–1.73, *p* = 0.04). Interestingly, this difference was more evident in the subgroups of Western Europe, whereas it was not significant in the subgroups from East Asia or Middle East.

In a population-based study involving 11,282 participants (14), the authors found that patients with Pso were at increased risk of having hyperuricemia compared with subjects without Pso (OR =1.37; *p* =0.04). Moreover, participants with Pso were more likely to develop gout (*OR* =1.83; *p* < 0.05) but both associations were no longer significant after adjustment for confounding factors.

However, contrasting results have been reported in literature. For instance, in a case-control study, Scott et al. (74) didn't find any significant difference in SUA levels between Pso patients, subjects with contact dermatitis and healthy individuals.

Additionally, other studies explored the relationship between UA and extent of cutaneous involvement in Pso. A cross-sectional study on 198 Pso patients found that SUA levels increased proportionally to the extent of cutaneous involvement in both genders and, in a multiple logistic regression model with hyperuricemia as dependent variable, PASI (Psoriasis Area Severity Index) score was a significant predictor (OR = 1.10; *p* = 0.03) (11). Similarly, Gisondi et al. (12) found that Pso patients with PASI score ≥10 exhibited higher SUA levels than subjects with less extensive skin disease. In a subsequent study (75), the same author found a prevalence of hyperuricemia of 20% in Pso patients, with higher SUA levels in obese individuals and no significant correlation with PASI. Furthermore, in a study by Ataseven et al. (76), PASI was weakly correlated with SUA (*r* = 0.27; *p* = 0.046). Conversely, in two cross-sectional studies (15, 16), SUA levels were not associated with the cutaneous extent of Pso. A *post-hoc* analysis of pooled data from three phase 3 trials with secukinumab, an IL-17A inhibitor, showed a statistically significant, although modest, association between the degree of skin involvement and SUA (*R*^2^ = 0.014; *p* < 0.0001). After 12 weeks of treatment, improvement in PASI score resulted in a decrease of SUA values (*R*^2^ = 0.014; *p* < 0.0001 univariately) (77). Finally, in a large population-based study on 39,111 patients with incident gout and 39,111 matched controls, the former had increased hazard ratios for developing Pso (HR =1.53, 95% CI 1.37–1.74; *p* < 0.05) and, in turn, Pso increased the risk of having incident gout (*OR* =1.32) (78).

Similar to Pso, the prevalence of hyperuricemia has been reported to be increased also in PsA, with estimates of 13.5% in men and 5% in women described by Lambert and Wright (79) and of 21% reported by Bruce et al. (80). Interestingly, in the latter study, no association between PASI and SUA was observed. In a multi-center, cross-sectional observational study of 160 Asian PsA patients, Lai et al. (82) found a prevalence of hyperuricemia of 31% and a significant correlation between hyperuricemia and PASI score (*p* = 0.05) or Body Surface Area (*p* = 0.04). Similar estimates were provided by AlJohani et al. (83) who described hyperuricemia in 31% of PsA patients in a prospective study. Moreover, compared to a normouricemic PsA control group, hyperuricemic patients had higher PASI scores (*p* = 0.006).

In a retrospective case–control study aimed to determine factors associated with development of PsA in subjects with psoriasis (84), hyperuricemia was a significant predictor after correcting for possible confounders (*OR* = 4.18; *p* < 0.01), thus suggesting that elevated SUA might represent a risk factor for the development of PsA in subjects with Pso.

However, not only the association between Pso or PsA and hyperuricemia has been investigated, but also the correlation between Pso or PsA and gout. In particular, in the subgroup of participants with confirmed Pso of a large, prospective population-based study (81), the multivariate-adjusted HR for gout were 2.72 (95% CI: 1.75, 4.25) in men and 1.40 (95% CI: 0.90, 2.19) in women. Interestingly, the risk of incident gout was substantially elevated in patients with Pso and concomitant PsA (HR = 4.95, 95% CI 2.72–9.01) and it was similar in males and females.

Furthermore, a case-control study in Taiwan (20) analyzed data of 114623 patients with gout compared with 114623 control subjects. Prevalence of Pso (1.6 vs. 1.1%; *p* < 0.0001) and PsA (0.3 vs. 0.1%; *p* < 0.0001) was higher in patients with gout than in controls. In multiple logistic regression analysis, gout was significantly associated with Pso (OR 1.30, 95% CI 1.20–1.42; *p* < 0.001) and PsA (OR 2.50, 95% CI 1.95–3.22; *p* < 0.001).

Finally, in a large retrospective observational study (21) including 14898 PsA patients, the risk for gout was significantly higher (incidence rate = 1.28 vs. 0.66; HR = 2.03; 95% CI 1.75–2.36) compared with the group of 35037 matched controls.

The association between Pso, PsA, and SUA levels has attracted the attention of researches since the first decades of 20th century (6, 7, 17–19). The reason behind this finding is not completely understood but probably the keratinocyte hyper-proliferation and increased cell turnover lead to enhanced catabolism of purines resulting in raised SUA. Furthermore, concomitant metabolic disorders such as metabolic syndrome, diabetes or obesity might contribute to the elevated UA levels observed in subjects with Pso. Over the years, a large number of studies has explored this topic, but the often-conflicting results don't allow to draw definitive conclusions.

## Uric Acid Levels and Cardiovascular Comorbidities in Psoriatic Arthritis

The cardiovascular consequences of hyperuricemia in PsA subjects were investigated by Gonzalez-Gay in a small observational study (85). To this purpose, 52 PsA patients without cardiovascular disease underwent clinical assessment with the aim to determine whether SUA was associated with ultrasound measures of subclinical atherosclerosis. Six individuals (11%) had hyperuricemia (defined as SUA >7 mg/dl) and were found to have a carotid intima-media thickness (IMT) greater than normo-uricemic patients (0.89 ± 0.20 vs. 0.67 ± 0.16 mm; *p* = 0.01). Furthermore, raised SUA levels were a risk factor for increased carotid IMT (*OR* = 2.66; *p* = 0.03) and for carotid plaques (*OR* =1.85; *p* = 0.05). However, these results should be interpreted with caution. Patients with hyperuricemia also had higher levels of serum creatinine, glucose, total cholesterol, and triglycerides. The authors tried to analyse the relationship between hyperuricemia and carotid IMT adjusting for these variables, but the small number of patients limited the analysis.

Similar results were obtained by Ibrahim et al. (86) comparing PsA patients with high SUA levels to those with normal values. The former had a significant increase in carotid IMT, presence of carotid plaques and impairment of flow-mediated dilation (FMD) (*p* < 0.001). In correlation analysis, SUA was positively associated with carotid IMT in PsA patients (*r* = 0.71; *p* < 0.001), with disease activity scores such as DAS-28 (*r* = 0.91; *p* < 0.001), and with PASI (*r* = 0.85; *p* < 0.001) and disease duration (*r* = 0.89; *p* < 0.001). Furthermore, a significant negative correlation with FMD of the brachial artery (*r* = −0.63; *p* < 0.001) was found.

## Effects of Anti-Rheumatic Drugs on Serum Uric Acid Levels in Psoriatic Arthritis

On the basis of the described association between PsA and UA, it can be hypothesized that the treatment with anti-rheumatic drugs could lower SUA levels. Some researchers had tested this hypothesis with conflicting results.

In a retrospective study, Wang et al. (87) explored this topic in a population of 99 patients with moderate to severe Pso treated with secukinumab for 24 weeks. Overall, after 24 weeks of treatment, patients showed significantly lower UA values compared to the baseline. Similar results were obtained in a *post-hoc* analysis of pooled data from three phase 3 studies with secukinumab (FIXTURE, ERASURE and SCULPTURE trials) in a population of patients with moderate to severe Pso (88). The total population was composed of 3,010 patients. After 52 weeks of treatment, UA levels were significantly reduced and cutaneous Pso had improved.

Unlike these reports, Karataş et al. (89) did not find significant differences in SUA levels after 6 months of follow-up in a small population of 36 patients (30 diagnosed with ankylosing spondylitis and 6 with PsA) receiving treatment with secukinumab.

Hasikova et al. (90) investigated the effect of TNF inhibitors on SUA levels in a population of patients with systemic autoimmune rheumatic diseases (rheumatoid arthritis, PsA, ankylosing spondylitis). Interestingly, after 3 months of treatments, the authors observed a significant increase in UA levels along with a reduction of inflammatory cytokines.

## Effects of Urate-Lowering Agents on Serum Inflammatory Mediators

From the above, it appears that subjects with Pso or PsA have a higher prevalence of hyperuricemia compared with the healthy population but another relevant point to explore is the effect of urate-lowering drugs on systemic inflammation.

In 1981, Goldman evaluated (19) this topic in a group of Pso patients treated with allopurinol. This study showed marked improvement of skin lesions in most cases, confirming the results of previous studies (91, 92). In more recent years, researchers investigated the effects of urate-lowering drugs on cytokines in patients with other inflammatory diseases such as colitis or gout (93, 94). Luis-Rodríguez et al. (95) determined the levels of serum CRP, TNF-α and IL-6 and assessed the mRNA expression of TNF-α and IL-6 in white blood cells of hyperuricemic patients and in subjects with normal SUA. The former had raised CRP and mRNA expression levels of both IL-6 and TNF-a (*p* < 0.001). Similar results were obtained also in multiple regression analysis. Finally, the inflammatory profile of a subgroup of 18 subjects was determined at baseline and after 6 months of treatment with allopurinol. The therapy decreased mRNA expression of TNF-α and IL-6 respectively by 23% and 52% (*p* < 0.001). Furthermore, a significant association was found between variations in SUA and changes in serum TNF-a (*r* = −0.62; *p* < 0.01) or IL-6 (*r* = −0.51; *p* < 0.05).

In a randomized, double-blind, placebo-controlled pilot study, Huang et al. (96) explored the effects of febuxostat on serum inflammatory markers such as IL-6, IL-17, and TNF-α in 156 Chinese patients with gout and hyperuricemia. Variables were measured at the baseline and at 2, 4, 8, 12, 16, and 24 weeks. After 8 weeks, treatment with febuxostat led to reduction of serum levels of IL-6, IL-17, and TNF-α by 38, 40, and 22%, respectively. The reduction in serum concentrations of pro-inflammatory cytokines correlated with the reduction of SUA levels.

In a similar study, Hao et al. (97) compared the effects of febuxostat and allopurinol in reducing serum levels of IL-1, IL-4, IL-6, IL-8, TNF-α, and cyclooxygenase-2 (COX-2) in 80 patients with gout, after 1 week and 3 months of treatment. Both treatments led to a significantly reduction of inflammatory cytokines, but febuxostat showed a more pronounced effect (*p* < 0.05). Furthermore, febuxostat led to marked reduction of cyclooxygenase-2 compared to allopurinol (*p* < 0.001).

The anti-inflammatory effects of urate-lowering agents were also studied in mice with induced colitis. The authors demonstrated that treatment with febuxostat led to a significant reduction of TNF-α, IL-1β, IL-6, and IFN-γ levels. In particular, it was observed a reduced expression of NF-kB in intestinal mucosa after treatment (98, 99). Finally, allopurinol was also used in the treatment of experimental autoimmune uveitis and it was shown to be more effective than prednisolone in suppressing the inflammatory reaction (100).

## Clinical Perspectives and Future Directions

PsA is a chronic systemic inflammatory disorder extending far beyond the involvement of skin and joints. This review dealt with the interplay between hyperuricemia and Pso/PsA, summarizing current knowledge on the topic and providing hints for future research. Analyzing the available literature, several issues regarding the mechanisms behind the association between UA and inflammation in Pso/PsA remain unresolved. Which factors contribute to hyperuricemia in Pso/PSA besides increased cellular turnover? What is the role of the kidney in this alteration? To what extent extra-renal organs, such as the intestine, are involved? Could the systemic inflammatory milieu stimulate UA production or hamper its secretion/excretion? Finally, could we still consider PsA and gout as two distinct entities or do they rather represent two facets of the same disease?

Current knowledge doesn't allow to draw firm conclusions about these points and future investigations are needed to shed new lights on this intricate field.

## Author Contributions

All authors listed have made a substantial, direct and intellectual contribution to the work, and approved it for publication.

## Conflict of Interest

The authors declare that the research was conducted in the absence of any commercial or financial relationships that could be construed as a potential conflict of interest.

## Publisher's Note

All claims expressed in this article are solely those of the authors and do not necessarily represent those of their affiliated organizations, or those of the publisher, the editors and the reviewers. Any product that may be evaluated in this article, or claim that may be made by its manufacturer, is not guaranteed or endorsed by the publisher.

## References

[1] Alinaghi F Calov M Kristensen LE Gladman DD Coates LC Jullien D. Prevalence of psoriatic arthritis in patients with psoriasis: a systematic review and meta-analysis of observational and clinical studies. J Am Acad Dermatol. (2019) 80:251–65.e19. 10.1016/j.jaad.2018.06.027 29928910

[2] Eder L Gladman DD. Psoriatic arthritis: phenotypic variance and nosology. Curr Rheumatol Rep. (2013) 15:316. 10.1007/s11926-013-0316-4 23371481

[3] Eder L Dey A Joshi AA Boehncke WH Mehta NN Szentpetery A. Cardiovascular diseases in psoriasis and psoriatic arthritis. J Rheumatol Suppl. (2019) 95:20–7. 10.3899/jrheum.190114 31154400

[4] Rahimi-Sakak F Maroofi M Rahmani J Bellissimo N Hekmatdoost A. Serum uric acid and risk of cardiovascular mortality: a systematic review and dose-response meta-analysis of cohort studies of over a million participants. BMC Cardiovasc Disord. (2019) 19:218. 10.1186/s12872-019-1215-z 31615412 PMC6792332

[5] Baumann RR Jillson OF. Hyperuricemia and psoriasis. J Invest Dermatol. (1961) 36:105–7. 13687990

[6] Eisen AZ Seegmiller JE. Uric acid metabolism in psoriasis. J Clin Invest. (1961) 40(8 Pt 1–2):1486–94. 10.1172/JCI104379 13726168 PMC292529

[7] Steinberg AG Becker SW Jr Fitzpatrick TB Kierland RR. A genetic and statistical study of psoriasis. Am J Hum Genet. (1951) 3:267–81. 14902766 PMC1716398

[8] Kamiya K Oiso N Kawada A Ohtsuki M. Epidemiological survey of the psoriasis patients in the Japanese Society for Psoriasis Research from 2013 to 2018. J Dermatol. (2021) 48:864–75. 10.1111/1346-8138.15803 33580908 PMC8247979

[9] Alpsoy S Akyuz A Erfan G Akkoyun DC Topcu B Guzel S. Atherosclerosis, some serum inflammatory markers in psoriasis. G Ital Dermatol Venereol. (2014) 149:167–75. 24819636

[10] Isha Jain VK Lal H. C-reactive protein and uric Acid levels in patients with psoriasis. Indian J Clin Biochem. (2011) 26:309–11. 10.1007/s12291-011-0132-4 22754198 PMC3162954

[11] Kwon HH Kwon IH Choi JW Youn JI. Cross-sectional study on the correlation of serum uric acid with disease severity in Korean patients with psoriasis. Clin Exp Dermatol. (2011) 36:473–8. 10.1111/j.1365-2230.2010.03988.x 21679368

[12] Gisondi P Targher G Cagalli A Girolomoni G. Hyperuricemia in patients with chronic plaque psoriasis. J Am Acad Dermatol. (2014) 70:127–30. 10.1016/j.jaad.2013.09.005 24183485

[13] Li X Miao X Wang H Wang Y Li F Yang Q. Association of serum uric acid levels in psoriasis: a systematic review and meta-analysis. Medicine. (2016) 95:e3676. 10.1097/MD.0000000000003676 27175702 PMC4902544

[14] Lai YC Yew YW. Psoriasis and uric acid: a population-based cross-sectional study. Clin Exp Dermatol. (2016) 41:260–6. 10.1111/ced.12781 26643816

[15] Solak B Dikicier BS Erdem T. Impact of elevated serum uric acid levels on systemic inflammation in patients with psoriasis. Angiology. (2017) 68:266–70. 10.1177/0003319716657980 27401209

[16] Gui XY Jin HZ Wang ZJ Xu TD. Serum uric acid levels and hyperuricemia in patients with psoriasis: a hospital-based cross-sectional study. An Bras Dermatol. (2018) 93:761–3. 10.1590/abd1806-4841.20187547 30156637 PMC6106671

[17] Lea WA Jr Curtis AC Bernstein IA. Serum uric acid levels in psoriasis. J Invest Dermatol. (1958) 31:269–71. 10.1038/jid.1958.119 13598933

[18] Tickner A Mier PD. Serum cholesterol, uric acid and proteins in psoriasis. Br J Dermatol. (1960) 72:132–7. 10.1111/j.1365-2133.1960.tb13860.x 13838320

[19] Goldman M. Uric acid in the etiology of psoriasis. Am J Dermatopathol. (1981) 3:397–404. 10.1097/00000372-198100340-00014 7337193

[20] Hu SC Lin CL Tu HP. Association between psoriasis, psoriatic arthritis and gout: a nationwide population-based study. J Eur Acad Dermatol Venereol. (2019) 33:560–7. 10.1111/jdv.15290 30317664

[21] Kaine J Song X Kim G Hur P Palmer JB. Higher incidence rates of comorbidities in patients with psoriatic arthritis compared with the general population using U.S. Administrative claims data. J Manag Care Spec Pharm. (2019) 25:122–32. 10.18553/jmcp.2018.17421 29694270 PMC10397587

[22] El Ridi R Tallima H. Physiological functions and pathogenic potential of uric acid: a review. J Adv Res. (2017) 8:487–93. 10.1016/j.jare.2017.03.003 28748115 PMC5512149

[23] Maiuolo J Oppedisano F Gratteri S Muscoli C Mollace V. Regulation of uric acid metabolism and excretion. Int J Cardiol. (2016) 213:8–14. 10.1016/j.ijcard.2015.08.109 26316329

[24] Stewart DJ Langlois V Noone D. Hyperuricemia and hypertension: links and risks. Integr Blood Press Control. (2019) 12:43–62. 10.2147/IBPC.S184685 31920373 PMC6935283

[25] Xu L Shi Y Zhuang S Liu N. Recent advances on uric acid transporters. Oncotarget. (2017) 8:100852–62. 10.18632/oncotarget.20135 29246027 PMC5725069

[26] Ichida K Matsuo H Takada T Nakayama A Murakami K Shimizu T. Decreased extra-renal urate excretion is a common cause of hyperuricemia. Nat Commun. (2012) 3:764. 10.1038/ncomms1756 22473008 PMC3337984

[27] Ben Salem C Slim R Fathallah N Hmouda H. Drug-induced hyperuricaemia and gout. Rheumatology. (2017) 56:679–88. 10.1093/rheumatology/kew293 27498351

[28] Pasalic D Marinkovic N Feher-Turkovic L. Uric acid as one of the important factors in multifactorial disorders–facts and controversies. Biochem Med. (2012) 22:63–75. 10.11613/BM.2012.007 22384520 PMC4062324

[29] Song Y Tang L Han J Gao Y Tang B Shao M. Uric acid provides protective role in red blood cells by antioxidant defense: a hypothetical analysis. Oxid Med Cell Longev. (2019) 2019:3435174. 10.1155/2019/3435174 31049132 PMC6458867

[30] Glantzounis GK Tsimoyiannis EC Kappas AM Galaris DA. Uric acid and oxidative stress. Curr Pharm Des. (2005) 11:4145–51. 10.2174/138161205774913255 16375736

[31] Ghaemi-Oskouie F Shi Y. The role of uric acid as an endogenous danger signal in immunity and inflammation. Curr Rheumatol Rep. (2011) 13:160–6. 10.1007/s11926-011-0162-1 21234729 PMC3093438

[32] Hara K Iijima K Elias MK Seno S Tojima I Kobayashi T. Airway uric acid is a sensor of inhaled protease allergens and initiates type 2 immune responses in respiratory mucosa. J Immunol. (2014) 192:4032–42. 10.4049/jimmunol.1400110 24663677 PMC4013745

[33] Rock KL Kataoka H Lai JJ. Uric acid as a danger signal in gout and its comorbidities. Nat Rev Rheumatol. (2013) 9:13–23. 10.1038/nrrheum.2012.143 22945591 PMC3648987

[34] Shi Y Evans JE Rock KL. Molecular identification of a danger signal that alerts the immune system to dying cells. Nature. (2003) 425:516–21. 10.1038/nature01991 14520412

[35] Shi Y Mucsi AD Ng G. Monosodium urate crystals in inflammation and immunity. Immunol Rev. (2010) 233:203–17. 10.1111/j.0105-2896.2009.00851.x 20193001

[36] Martinon F. Update on biology: uric acid and the activation of immune and inflammatory cells. Curr Rheumatol Rep. (2010) 12:135–41. 10.1007/s11926-010-0092-3 20425023

[37] Alvarez-Lario B Macarrón-Vicente J. Is there anything good in uric acid? QJM. (2011) 104:1015–24. 10.1093/qjmed/hcr159 21908382

[38] Brovold H Lund T Svistounov D Solbu MD Jenssen TG Ytrehus K. Crystallized but not soluble uric acid elicits pro-inflammatory response in short-term whole blood cultures from healthy men. Sci Rep. (2019) 9:10513. 10.1038/s41598-019-46935-w 31324844 PMC6642259

[39] Mentis AA Dardiotis E Efthymiou V Chrousos GP. Non-genetic risk and protective factors and biomarkers for neurological disorders: a meta-umbrella systematic review of umbrella reviews. BMC Med. (2021) 19:6. 10.1186/s12916-020-01873-7 33435977 PMC7805241

[40] Cortese M Riise T Engeland A Ascherio A Bjørnevik K. Urate and the risk of Parkinson's disease in men and women. Parkinsonism Relat Disord. (2018) 52:76–82. 10.1016/j.parkreldis.2018.03.026 29615298

[41] Shen C Guo Y Luo W Lin C Ding M. Serum urate and the risk of Parkinson's disease: results from a meta-analysis. Can J Neurol Sci. (2013) 40:73–9. 10.1017/S0317167100012981 23250131

[42] Boccardi V Carino S Marinelli E Lapenna M Caironi G Bianco AR. Uric acid and late-onset Alzheimer's disease: results from the ReGAl 2.0 project. Aging Clin Exp Res. (2021) 33:361–6. 10.1007/s40520-020-01541-z 32277437

[43] Zhou Z Zhong S Liang Y Zhang X Zhang R Kang K. Serum uric acid and the risk of dementia: a systematic review and meta-analysis. Front Aging Neurosci. (2021) 13:625690. 10.3389/fnagi.2021.625690 33716713 PMC7947796

[44] Wang YF Li JX Sun XS Lai R Sheng WL. High serum uric acid levels are a protective factor against unfavourable neurological functional outcome in patients with ischaemic stroke. J Int Med Res. (2018) 46:1826–38. 10.1177/0300060517752996 29529907 PMC5991245

[45] Cheng GM Wang RL Zhang B Deng XY. The protective effect of uric acid in reducing TLR4/NF-κB activation through the inhibition of HMGB1 acetylation in a model of ischemia-reperfusion injury *in vitro*. Mol Biol Rep. (2020) 47:3233–40. 10.1007/s11033-020-05324-7 32095984

[46] Li X Meng X Timofeeva M Tzoulaki I Tsilidis KK Ioannidis JP. Serum uric acid levels and multiple health outcomes: umbrella review of evidence from observational studies, randomised controlled trials, and Mendelian randomisation studies. BMJ. (2017) 357:j2376. 10.1136/bmj.j2376 28592419 PMC5461476

[47] Aroor AR Demarco VG Jia G Sun Z Nistala R Meininger GA. The role of tissue Renin-Angiotensin-aldosterone system in the development of endothelial dysfunction and arterial stiffness. Front Endocrinol. (2013) 4:161. 10.3389/fendo.2013.00161 24194732 PMC3810594

[48] Aroor AR Jia G Habibi J Sun Z Ramirez-Perez FI Brady B. Uric acid promotes vascular stiffness, maladaptive inflammatory responses and proteinuria in western diet fed mice. Metabolism. (2017) 74:32–40. 10.1016/j.metabol.2017.06.006 28764846 PMC5577816

[49] Ghasemi A. Uric acid-induced pancreatic β-cell dysfunction. BMC Endocr Disord. (2021) 21:24. 10.1186/s12902-021-00698-6 33593356 PMC7888074

[50] Ponticelli C Podestà MA Moroni G. Hyperuricemia as a trigger of immune response in hypertension and chronic kidney disease. Kidney Int. (2020) 98:1149–59. 10.1016/j.kint.2020.05.056 32650020

[51] Li J Li X Hou R Liu R Zhao X Dong F. Psoriatic T cells reduce epidermal turnover time and affect cell proliferation contributed from differential gene expression. J Dermatol. (2015) 42:874–80. 10.1111/1346-8138.12961 26046687

[52] Beringer A Miossec P. Systemic effects of IL-17 in inflammatory arthritis. Nat Rev Rheumatol. (2019) 15:491–501. 10.1038/s41584-019-0243-5 31227819

[53] Szendroedi J Chmelik M Schmid AI Nowotny P Brehm A Krssak M. Abnormal hepatic energy homeostasis in type 2 diabetes. Hepatology. (2009) 50:1079–86. 10.1002/hep.23093 19637187

[54] Cortez-Pinto H Chatham J Chacko VP Arnold C Rashid A Diehl AM. Alterations in liver ATP homeostasis in human nonalcoholic steatohepatitis: a pilot study. JAMA. (1999) 282:1659–64. 10.1001/jama.282.17.1659 10553793

[55] Petrie JL Patman GL Sinha I Alexander TD Reeves HL Agius L. The rate of production of uric acid by hepatocytes is a sensitive index of compromised cell ATP homeostasis. Am J Physiol Endocrinol Metab. (2013) 305:E1255–65. 10.1152/ajpendo.00214.2013 24045866

[56] Hosomi A Nakanishi T Fujita T Tamai I. Extra-renal elimination of uric acid via intestinal efflux transporter BCRP/ABCG2. PLoS ONE. (2012) 7:e30456. 10.1371/journal.pone.0030456 22348008 PMC3277506

[57] Fieldhouse KA Ukaibe S Crowley EL Khanna R O'Toole A Gooderham MJ. Inflammatory bowel disease in patients with psoriasis treated with interleukin-17 inhibitors. Drugs Context. (2020) 9. 10.7573/dic.2020-2-1 32362930 PMC7185907

[58] Takada T Ichida K Matsuo H Nakayama A Murakami K Yamanashi Y. ABCG2 dysfunction increases serum uric acid by decreased intestinal urate excretion. Nucleosides Nucleotides Nucleic Acids. (2014) 33:275–81. 10.1080/15257770.2013.854902 24940679

[59] Englund G Jacobson A Rorsman F Artursson P Kindmark A Rönnblom A. Efflux transporters in ulcerative colitis: decreased expression of BCRP. (ABCG2) and Pgp. (ABCB1). Inflamm Bowel Dis. (2007) 13:291–7. 10.1002/ibd.20030 17206689

[60] Perez-Chada LM Merola JF. Comorbidities associated with psoriatic arthritis: Review and update. Clin Immunol. (2020) 214:108397. 10.1016/j.clim.2020.108397 32229290

[61] Gong M Wen S Nguyen T Wang C Jin J Zhou L. Converging relationships of obesity and hyperuricemia with special reference to metabolic disorders and plausible therapeutic implications. Diabetes Metab Syndr Obes. (2020) 13:943–62. 10.2147/DMSO.S232377 32280253 PMC7125338

[62] Quiñones-Galvan A Ferrannini E. Renal effects of insulin in man. J Nephrol. (1997) 10:188–91. 9377725

[63] Matsuura F Yamashita S Nakamura T Nishida M Nozaki S Funahashi T. Effect of visceral fat accumulation on uric acid metabolism in male obese subjects: visceral fat obesity is linked more closely to overproduction of uric acid than subcutaneous fat obesity. Metabolism. (1998) 47:929–33. 10.1016/S0026-0495(98)90346-8 9711987

[64] Toyoki D Shibata S Kuribayashi-Okuma E Xu N Ishizawa K Hosoyamada M. Insulin stimulates uric acid reabsorption via regulating urate transporter 1 and ATP-binding cassette subfamily G member 2. Am J Physiol Renal Physiol. (2017) 313:F826–34. 10.1152/ajprenal.00012.2017 28679589

[65] Felten R Duret PM Gottenberg JE Spielmann L Messer L. At the crossroads of gout and psoriatic arthritis: “psout”. Clin Rheumatol. (2020) 39:1405–13. 10.1007/s10067-020-04981-0 32062768

[66] Oliviero F Scanu A Galozzi P Gava A Frallonardo P Ramonda R. Prevalence of calcium pyrophosphate and monosodium urate crystals in synovial fluid of patients with previously diagnosed joint diseases. Joint Bone Spine. (2013) 80:287–90. 10.1016/j.jbspin.2012.08.006 23021157

[67] Raucci F Iqbal AJ Saviano A Minosi P Piccolo M Irace C. IL-17A neutralizing antibody regulates monosodium urate crystal-induced gouty inflammation. Pharmacol Res. (2019) 147:104351. 10.1016/j.phrs.2019.104351 31315067

[68] Klück V Liu R Joosten LAB. The role of interleukin-1 family members in hyperuricemia and gout. Joint Bone Spine. (2021) 88:105092. 10.1016/j.jbspin.2020.105092 33129923

[69] Uratsuji H Tada Y Hau CS Shibata S Kamata M Kawashima T. Monosodium urate crystals induce functional expression of P2Y14 receptor in human keratinocytes. J Invest Dermatol. (2016) 136:1293–6. 10.1016/j.jid.2016.01.026 26872603

[70] Ondet T Muscatelli-Groux B Coulouarn C Robert S Gicquel T Bodin A. The release of pro-inflammatory cytokines is mediated via mitogen-activated protein kinases rather than by the inflammasome signalling pathway in keratinocytes. Clin Exp Pharmacol Physiol. (2017) 44:827–38. 10.1111/1440-1681.12765 28425217

[71] Uratsuji H Tada Y Kawashima T Kamata M Hau CS Asano Y. P2Y6 receptor signaling pathway mediates inflammatory responses induced by monosodium urate crystals. J Immunol. (2012) 188:436–44. 10.4049/jimmunol.1003746 22102722

[72] Zamudio-Cuevas Y Fernández-Torres J Martínez-Nava GA Martínez-Flores K Ramírez Olvera A Medina-Luna D. Phagocytosis of monosodium urate crystals by human synoviocytes induces inflammation. Exp Biol Med. (2019) 244:344–51. 10.1177/1535370219830665 30739483 PMC6488864

[73] Chen DP Wong CK Tam LS Li EK Lam CW. Activation of human fibroblast-like synoviocytes by uric acid crystals in rheumatoid arthritis. Cell Mol Immunol. (2011) 8:469–78. 10.1038/cmi.2011.35 21946433 PMC4012929

[74] Scott JT Stodell MA. Serum uric acid levels in psoriasis. Adv Exp Med Biol. (1984) 165 Pt A:283–5. 10.1007/978-1-4684-4553-4_55 6720391

[75] Gisondi P. Hyperuricemia in patients with chronic plaque psoriasis. Drug Dev Res. (2014) 75(Suppl 1):S70–2. 10.1002/ddr.21201 25381984

[76] Ataseven A Kesli R Kurtipek GS Ozturk P. Assessment of lipocalin 2, clusterin, soluble tumor necrosis factor receptor-1, interleukin-6, homocysteine, and uric acid levels in patients with psoriasis. Dis Markers. (2014) 2014:541709. 10.1155/2014/541709 24803721 PMC3996950

[77] Dehlin M Fasth AER Reinhardt M Jacobsson LTH. Impact of psoriasis disease activity and other risk factors on serum urate levels in patients with psoriasis and psoriatic arthritis-a post-hoc analysis of pooled data from three phase 3 trials with secukinumab. Rheumatol Adv Pract. (2021) 5:rkab009. 10.1093/rap/rkab009 33748660 PMC7962727

[78] Kuo CF Grainge MJ Mallen C Zhang W Doherty M. Comorbidities in patients with gout prior to and following diagnosis: case-control study. Ann Rheum Dis. (2016) 75:210–7. 10.1136/annrheumdis-2014-206410 25398375 PMC4717388

[79] Lambert JR Wright V. Serum uric acid levels in psoriatic arthritis. Ann Rheum Dis. (1977) 36:264–67. 10.1136/ard.36.3.264 879864 PMC1006678

[80] Bruce IN Schentag CT Gladman DD. Hyperuricemia in psoriatic arthritis: prevalence and associated features. J Clin Rheumatol. (2000) 6:6–9. 10.1097/00124743-200002000-00001 19078442

[81] Merola JF Wu S Han J Choi HK Qureshi AA. Psoriasis, psoriatic arthritis and risk of gout in US men and women. Ann Rheum Dis. (2015) 74:1495–500. 10.1136/annrheumdis-2014-205212 24651620 PMC4224633

[82] Lai TL Yim CW Wong PY Leung MC Ng WL. Hyperuricemia in Asian psoriatic arthritis patients. Int J Rheum Dis. (2018) 21:843–9. 10.1111/1756-185X.13265 29349920

[83] AlJohani R Polachek A Ye JY Chandran V Gladman DD. Characteristic and outcome of psoriatic arthritis patients with hyperuricemia. J Rheumatol. (2018) 45:213–7. 10.3899/jrheum.170384 29196385

[84] Tsuruta N Imafuku S Narisawa Y. Hyperuricemia is an independent risk factor for psoriatic arthritis in psoriatic patients. J Dermatol. (2017) 44:1349–52. 10.1111/1346-8138.13968 28691207

[85] Gonzalez-Gay MA Gonzalez-Juanatey C Vazquez-Rodriguez TR Gomez-Acebo I Miranda-Filloy JA Paz-Carreira J. Asymptomatic hyperuricemia and serum uric acid concentration correlate with subclinical atherosclerosis in psoriatic arthritis patients without clinically evident cardiovascular disease. Semin Arthritis Rheum. (2009) 39:157–62. 10.1016/j.semarthrit.2008.06.001 18722649

[86] Ibrahim SE Helmi A Yousef TM Hassan MS Farouk N. Association of asymptomatic hyperuricemia and endothelial dysfunction in psoriatic arthritis. Egyptian Rheumatologist. (2012) 34:83–9. 10.1016/j.ejr.2012.03.002

[87] Wang HN Huang YH. Changes in metabolic parameters in psoriatic patients treated with secukinumab. Ther Adv Chronic Dis. (2020) 11:2040622320944777. 10.1177/2040622320944777 32821362 PMC7412909

[88] Gerdes S Pinter A Papavassilis C Reinhardt M. Effects of secukinumab on metabolic and liver parameters in plaque psoriasis patients. J Eur Acad Dermatol Venereol. (2020) 34:533–41. 10.1111/jdv.16004 31599476 PMC7065121

[89] Karataş A Gerçek AN Öz B Gözel N Pişkin Sagir R Gür M. The effect of secukinumab treatment on hematological parameters in ankylosing spondylitis and psoriatic arthritis. Eur J Rheumatol. (2020) 7:169–72. 10.5152/eurjrheum.2020.20109 32910771 PMC7574766

[90] Hasikova L Pavlikova M Hulejova H Kozlik P Kalikova K Mahajan A. Serum uric acid increases in patients with systemic autoimmune rheumatic diseases after 3 months of treatment with TNF inhibitors. Rheumatol Int. (2019) 39:1749–57. 10.1007/s00296-019-04394-6 31363829

[91] Viglioglia PA Plante GE Viglioglia J Saraceno EF. Allopurinol in psoriasis. Dermatologica. (1970) 141:203–7. 10.1159/000252467 4924504

[92] Feuerman EJ Nir MA. Allopurinol in psoriasis–a double-blind study. Br J Dermatol. (1973) 89:83–6. 10.1111/j.1365-2133.1973.tb01921.x 4274726

[93] Kondratiuk VE Tarasenko OM Karmazina OM Taranchuk VV. Impact of the synbiotics and urate-lowering therapy on gut microbiota and cytokine profile in patients with chronic gouty arthritis. J Med Life. (2020) 13:490–8. 10.25122/jml-2020-0065 33456597 PMC7803318

[94] Alghamdi YS Soliman MM Nassan MA. Impact of Lesinurad and allopurinol on experimental Hyperuricemia in mice: biochemical, molecular and Immunohistochemical study. BMC Pharmacol Toxicol. (2020) 21:10. 10.1186/s40360-020-0386-7 32041665 PMC7011467

[95] Luis-Rodríguez D Donate-Correa J Martín-Núñez E Ferri C Tagua VG Pérez Castro A. Serum urate is related to subclinical inflammation in asymptomatic hyperuricaemia. Rheumatology. (2021) 60:371–9. 10.1093/rheumatology/keaa425 32901294

[96] Huang YY Ye Z Gu SW Jiang ZY Zhao L. The efficacy and tolerability of febuxostat treatment in a cohort of Chinese Han population with history of gout. J Int Med Res. (2020) 48:300060520902950. 10.1177/0300060520902950 32363973 PMC7221481

[97] Hao G Duan W Sun J Liu J Peng B. Effects of febuxostat on serum cytokines IL-1, IL-4, IL-6, IL-8, TNF-α and COX-2. Exp Ther Med. (2019) 17:812–6. 10.3892/etm.2018.6972 30651867 PMC6307400

[98] Amirshahrokhi K. Febuxostat attenuates ulcerative colitis by the inhibition of NF-κB, proinflammatory cytokines, and oxidative stress in mice. Int Immunopharmacol. (2019) 76:105884. 10.1016/j.intimp.2019.105884 31499267

[99] El-Mahdy NA Saleh DA Amer MS Abu-Risha SE. Role of allopurinol and febuxostat in the amelioration of dextran-induced colitis in rats. Eur J Pharm Sci. (2020) 141:105116. 10.1016/j.ejps.2019.105116 31654756

[100] Namazi MR. Cannabinoids, loratadine and allopurinol as novel additions to the antipsoriatic ammunition. J Eur Acad Dermatol Venereol. (2005) 19:319–22. 10.1111/j.1468-3083.2004.01184.x 15857457

